# Nationwide survey on neonatal resuscitation across delivery facilities in Japan

**DOI:** 10.1111/ped.70335

**Published:** 2026-02-05

**Authors:** Hasumi Tomita, Takahiro Sugiura, Hitomi Arahori, Shunsuke Tamaru, Kimiko Enomoto, Eiji Hirakawa, Masaki Wada, Isao Kusakawa, Tetsuya Isayama, Tomomi Kotani, Shigeharu Hosono

**Affiliations:** ^1^ Department of Obstetrics and Gynecology Tohoku University Hospital Sendai Japan; ^2^ Department of Pediatrics and Neonatology Nagoya City University Graduate School of Medical Sciences Nagoya Japan; ^3^ Department of Pediatrics Toyonaka Municipal Hospital Osaka Japan; ^4^ Department of Obstetrics and Gynecology Saitama Medical University Saitama Japan; ^5^ Department of Obstetrics and Gynecology Odawara Municipal Hospital Odawara Japan; ^6^ Department of Neonatology Kagoshima City Hospital Kagoshima Japan; ^7^ Department of Health and Welfare Niigata Prefecture Niigata Japan; ^8^ Department Human Life Sciences Tokyo City University Tokyo Japan; ^9^ Department of Neonatology National Center for Child Health and Development Tokyo Japan; ^10^ Department of Obstetrics and Gynecology Hamamatsu University School of Medicine Hamamatsu Shizuoka Japan; ^11^ Department of Pediatrics (Neonatology), Nerima Hikarigaoka Hospital Japan Community Health Care Organization (JCHO) Tokyo Japan

**Keywords:** equipment availability, national survey, neonatal resuscitation, supraglottic airway devices, umbilical cord management

## Abstract

**Background:**

Neonatal resuscitation is crucial for transitioning to extrauterine life. The Neonatal Cardiopulmonary Resuscitation (NCPR) guidelines in Japan were first developed based on the 2005 International Liaison Committee on Resuscitation (ILCOR) recommendations and have since been revised approximately every 5 years. However, the extent of their implementation in clinical practice remains unclear. This study aimed to assess the current status of neonatal resuscitation practices.

**Methods:**

We conducted a survey targeting 2297 delivery facilities in Japan. The questionnaire assessed equipment availability, provider training, and adoption of updated practices.

**Results:**

In total, 1553 facilities responded, of which 1505 were actively conducting deliveries. Therefore, the analysis was performed based on the responses from 1505 delivery‐performing facilities. Pulse oximeters were widely available, whereas electrocardiogram monitors and oxygen–air blenders were less common. T‐piece resuscitators were used in 32% of the facilities. Only 11% of the facilities had experience using supraglottic airway devices. Although 81% reported that all or almost all staff had completed NCPR training, 23 clinics lacked trained personnel. Regarding umbilical cord management in infants born before 28 weeks of gestation, cut cord milking was the most common method, followed by intact cord milking, early cord clamping, and delayed cord clamping.

**Conclusions:**

Neonatal resuscitation systems are generally established across delivery facilities in Japan. However, the implementation of revised guidelines varies based on facility type. These findings offer important insights for the upcoming 2025 NCPR guideline revision and emphasize the need for targeted training and equitable resource distribution to improve neonatal outcomes.

## INTRODUCTION

The adaptation to extrauterine respiration is a critical transition that occurs rapidly after birth. Although oxygenation during fetal life depends on the placenta, newborns must clear their lung fluid, begin spontaneous breathing, and establish pulmonary circulation after birth. Although most term infants breathe spontaneously within 30 s after birth, some require interventions, such as stimulation, ventilation, or advanced resuscitation measures.^1^, ^2^, ^3^, ^4^, ^5^ As it is often unpredictable which newborns require intervention, all delivery settings must be prepared to provide immediate neonatal resuscitation.

To improve neonatal outcomes, the International Liaison Committee on Resuscitation (ILCOR) regularly updates evidence‐based recommendations (Consensus on Science with Treatment Recommendations [CoSTRs]) that guide national resuscitation guidelines.^6^ In Japan, neonatal resuscitation education was formally introduced in 2007 with the launch of the Neonatal Cardiopulmonary Resuscitation (NCPR) course, developed based on the 2005 ILCOR CoSTR and led by the Japan Society of Perinatal and Neonatal Medicine. Subsequently, national guidelines were revised in alignment with the 2010, 2015, and 2020 ILCOR CoSTR updates.^7^, ^8^, ^9^, ^10^ The ILCOR emphasizes the need for the contextual adaptation of recommendations, highlighting the importance of tailoring neonatal resuscitation practices to local healthcare systems, resource availability, cultural preferences, and training environments.^6^ This underscores the necessity for gathering up‐to‐date Japan‐specific data in preparation for the anticipated 2025 revision of the NCPR guidelines.

Although nationwide surveys on neonatal resuscitation practices were conducted in 2005, 2010, 2013, and 2015, all preceded the two major revisions of the NCPR guidelines that followed the corresponding CoSTR updates.^11^, ^12^, ^13^, ^14^ These surveys evaluated the availability of equipment such as pulse oximeters and oxygen–air blenders, but did not address the use of ECG monitors, T‐piece resuscitators, supraglottic airway (SGA) devices, or cord management practices. Since then, several key changes have emerged in the CoSTR, including strengthened recommendations for electrocardiogram (ECG) monitoring, recognition of the advantages of T‐piece resuscitators in providing positive pressure ventilation (PPV), endorsement of supraglottic airway (SGA) devices as backup options when intubation or PPV is difficult, and updated guidance on cord management, particularly the utility of delayed cord clamping (DCC) in preterm infants. In the Japanese NCPR guidelines, the 2015 revision introduced ECG monitoring as a consideration in the resuscitation algorithm and added a commentary on cord management practices.^7^

However, the effect of these CoSTR updates on neonatal resuscitation practices in Japan remains unclear. Thus, there is a pressing need to evaluate current practices, with particular attention to these specific components. Approximately half of all deliveries in Japan occur in small maternity clinics, where pediatricians are not on duty 24 h a day.^15^

We conducted a nationwide survey of delivery facilities in Japan to assess the current status of neonatal resuscitation practices. These findings are expected to inform future policy developments, improve training programs, and guide revisions of resuscitation protocols. Moreover, the data will serve as a foundational resource for the successful revision and implementation of the 2025 NCPR guidelines.

## METHODS

### Study design

This study used a cross‐sectional design to evaluate the status of neonatal resuscitation practices in various healthcare facilities in Japan.

### Study population and setting

A questionnaire was sent to medical institutions registered with the following three societies to ascertain the delivery of medical institutions in Japan. Duplicate registered institutions were screened and sorted out:

1. Birthing facilities registered with the Japan Society of Obstetrics and Gynecology
2. Perinatal centers responsible for neonatal resuscitation are registered with the Japan Society of Perinatal and Neonatal Medicine
3. Birth centers handling deliveries registered with the Japanese Midwives Association

Healthcare facilities were categorized into five types according to their roles and perinatal care capabilities:

1. *Comprehensive perinatal care centers (Comprehensive PCCs)*: Tertiary‐level centers providing advanced care for high‐risk pregnancies, severe obstetric complications, and neonatal intensive care and staffed 24 h a day by specialized medical teams and nurses.^16^
2. *Regional perinatal care Centers (Regional PCCs)*: Secondary‐level facilities managing moderate‐ to high‐risk pregnancies and neonatal stabilization by collaborating closely with comprehensive PCCs.
3. *Non‐PCC hospitals*: General hospitals primarily handled low‐to‐moderate‐risk pregnancies by referring high‐risk cases to perinatal centers.
4. *Obstetric clinics*: Smaller, typically private facilities for routine antenatal care and low‐risk deliveries, lacking neonatal intensive care capability and relying on referrals for emergencies.
5. *Midwifery centers*: Independent centers operated by midwives managing only low‐risk deliveries without medical complications (emergency transfer systems are critical because advanced care is unavailable on‐site).

### Data collection

The questionnaire comprised 38 items designed to assess neonatal resuscitation practices across Japanese birthing facilities, covering topics such as facility characteristics, availability and use of resuscitation equipment, personnel responsibilities and training, implementation of guideline‐recommended practices (e.g., thermal management, DCC, use of ECG and SGA devices), and attitudes toward telemedicine.

The questionnaire was administered using Google Forms, a secure web‐based survey platform provided by Google LLC. The survey included items regarding facility characteristics or types, temperature management, neonatal resuscitation personnel and training, device availability, PPV practice, SGA use, umbilical cord management, and other related aspects. The full content of the questionnaire is shown in Appendix S1. Invitations with a URL link and QR codes to access the survey were distributed to eligible facilities by post in early August 2024, accompanied by a detailed explanatory document outlining the study's objectives and procedures. Participation in this study was voluntary. A reminder postcard was sent in September 2024, and the survey remained open until September 30, 2024.

### Data analysis

Descriptive statistics were used to summarize the survey responses. The results are presented as the numbers and percentages of respondents for each item. For each question, the percentages shown in the main text and figures were calculated based on the number of facilities that provided valid responses, excluding nonresponders from the denominator. Statistical analyses were not performed.

### Ethical considerations

This study did not collect sensitive personal data and focused solely on facility‐level information. Ethical approval was obtained from the Ethics Committee of Toyohashi Municipal Hospital (approval no. 819). Informed consent was deemed unnecessary as no individual‐level data were collected and participation in this survey was voluntary.

## RESULTS

Survey invitations were sent to 2296 facilities, of which 1553 responded (response rate, 68%). Among these, 1505 facilities were actively conducting deliveries at the time of response, including 110 comprehensive PCCs, 242 regional PCCs, 341 non‐PCC hospitals, 580 obstetric clinics, and 228 midwifery centers. A comprehensive summary of the number of responses and corresponding percentages for all survey items, including those with missing responses, is provided in Table S1.

### Equipment for neonatal resuscitation

**FIGURE 1:**
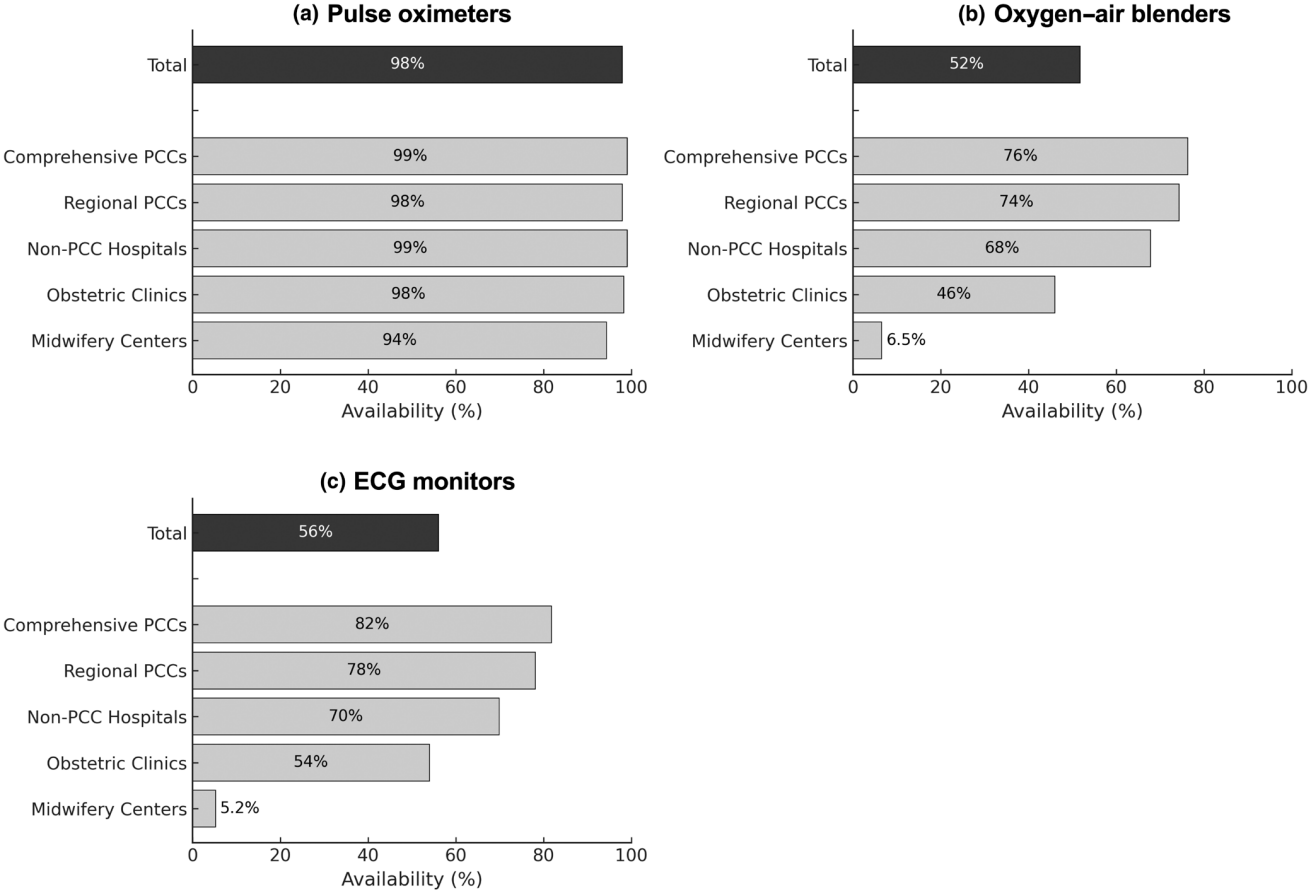
Availability of pulse oximeters, oxygen–air blenders, and electrocardiogram (ECG) monitors based on facility type. Availability of pulse oximeters, oxygen–air blenders, and ECG monitors for neonatal resuscitation, stratified based on facility type. Each bar represents the percentage of facilities reporting the availability of each device. A total of 1505 facilities responded, including Comprehensive PCCs (*n* = 110), Regional PCCs (*n* = 242), Non‐PCC hospitals (*n* = 342), Obstetric Clinics (*n* = 581), and Midwifery Centers (*n* = 230).

Pulse oximeters were widely available, with an overall installation rate of 98%. Oxygen–air blenders were present in 52% of the facilities. Availability varied based on facility type, with obstetric clinics and midwifery centers reporting lower rates (46% and 6.5%, respectively). ECG monitors were available in 56% of the facilities, with 54% of obstetric clinics and only 5.2% of midwifery centers equipped with them (Figure 1).

### Healthcare professionals responsible for neonatal resuscitation

**FIGURE 2:**
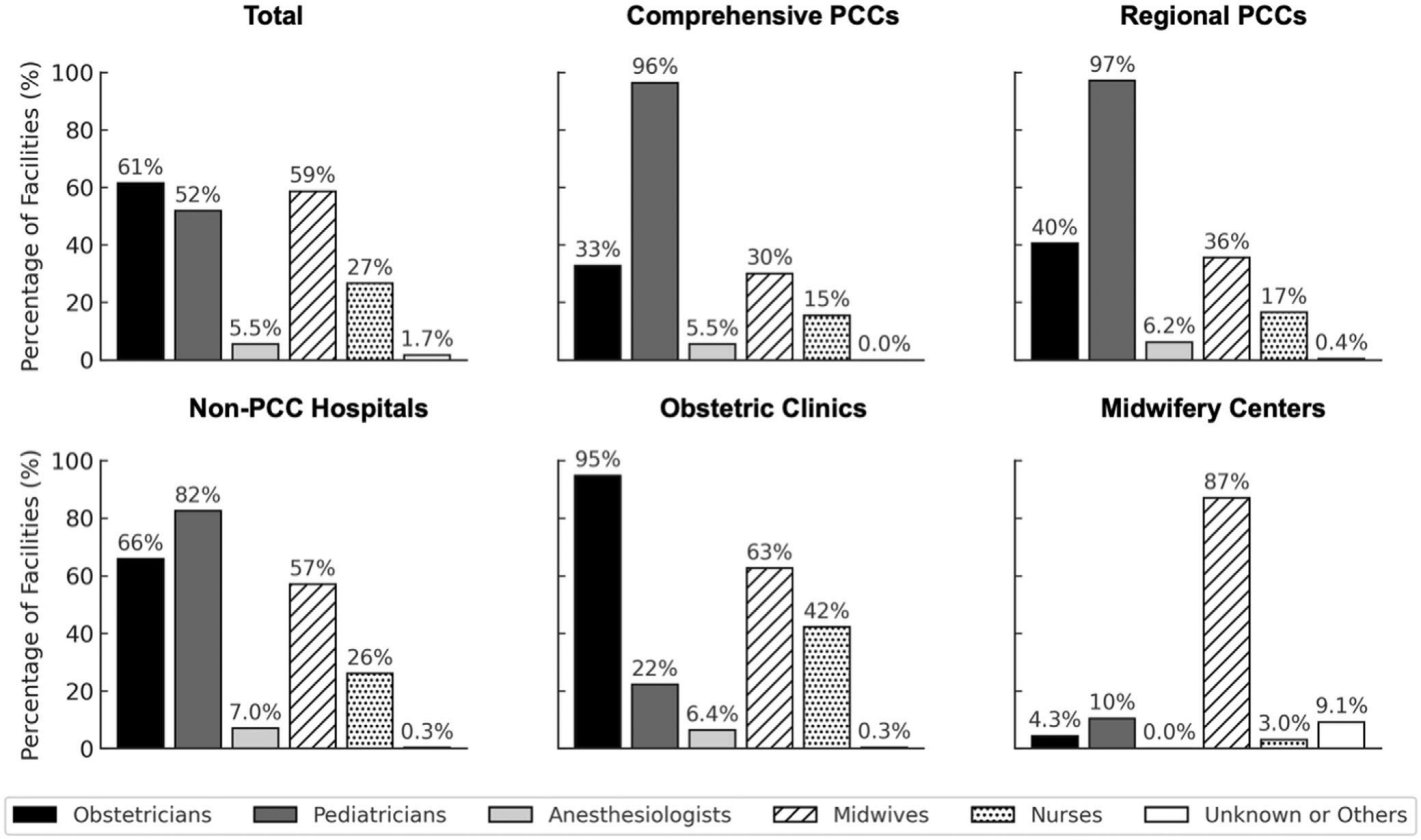
Primary providers of neonatal resuscitation when interventions beyond initial stabilization were anticipated. Distribution of healthcare professionals responsible for neonatal resuscitation beyond initial stabilization, stratified based on facility type. The respondents were allowed to select multiple options. Obstetricians, pediatricians, anesthesiologists, midwives, nurses, and other providers are represented as percentages of all responding facilities within each category: Total (*n* = 1497), Comprehensive PCCs (*n* = 110), Regional PCCs (*n* = 242), Non‐PCC hospitals (*n* = 342), Obstetric Clinics (*n* = 578), and Midwifery Centers (*n* = 225).

Obstetricians and midwives were most commonly involved in neonatal resuscitation beyond initial stabilization, reported in 62% and 59% of the facilities, respectively. Pediatricians were the main providers of comprehensive and regional PCCs (96% and 97%, respectively). In contrast, obstetricians played a central role in non‐PCC hospitals (66%) and obstetric clinics (95%). Midwives were responsible for 87% of the midwifery centers (Figure 2).

**FIGURE 3:**
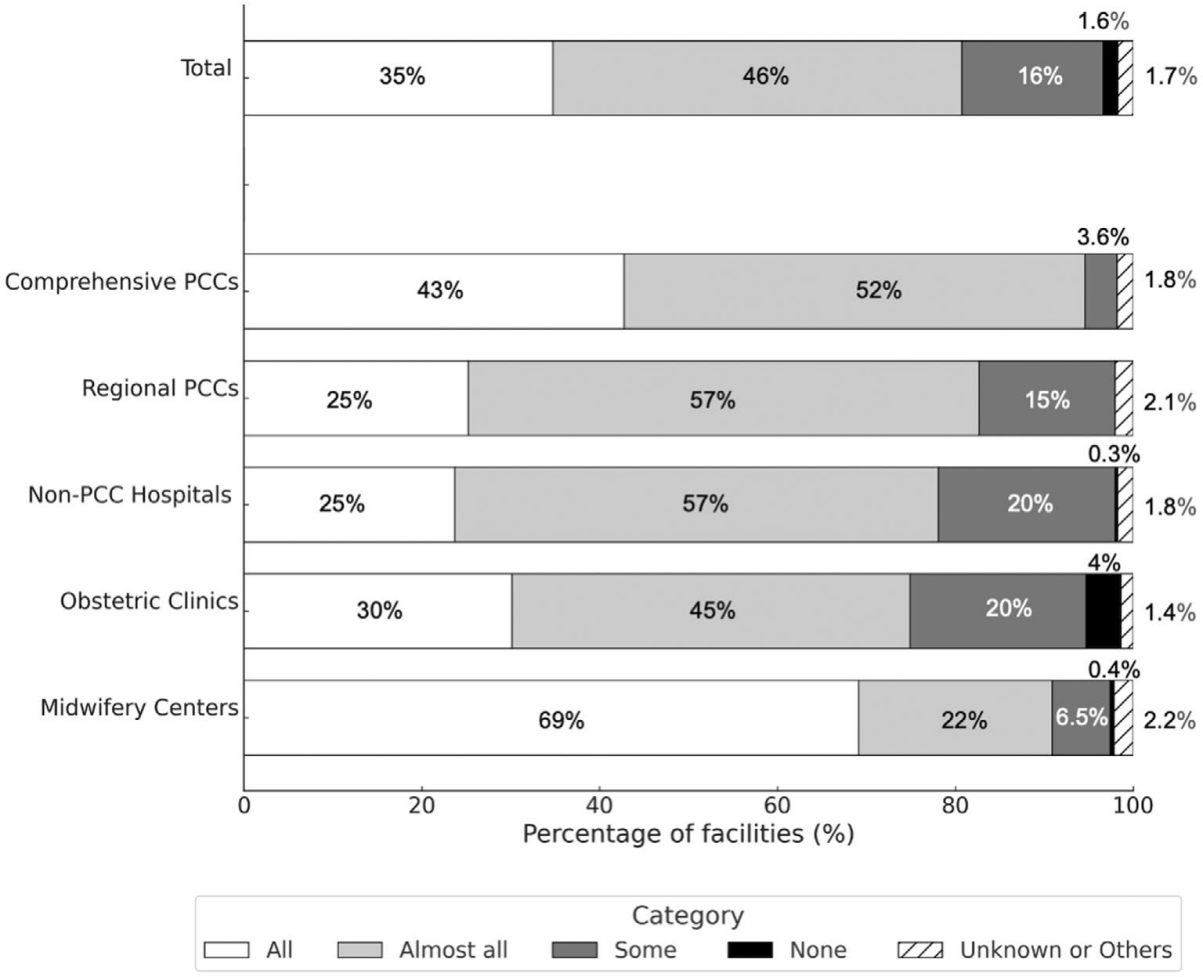
Proportion of facilities in which neonatal resuscitation providers had completed Neonatal Cardiopulmonary Resuscitation (NCPR) training. Distribution of facilities based on the proportion of healthcare professionals responsible for neonatal resuscitation who had completed NCPR training, stratified based on facility type. Response options included the following: “All,” “Almost all,” “Some,” “None,” and “Unknown or Others.” A total of 1505 facilities responded, including Comprehensive PCCs (*n* = 110), Regional PCCs (*n* = 242), Non‐PCC Hospitals (*n* = 342), Obstetric Clinics (*n* = 581), and Midwifery Centers (*n* = 230).

The proportion of facilities where all or almost all providers had completed NCPR training was 81%. In contrast, 1.7% of the facilities reported that none of the providers had undergone training. This included one hospital, 23 obstetric clinics, and one midwifery center (Figure 3).

### Methods of positive pressure ventilation

**FIGURE 4:**
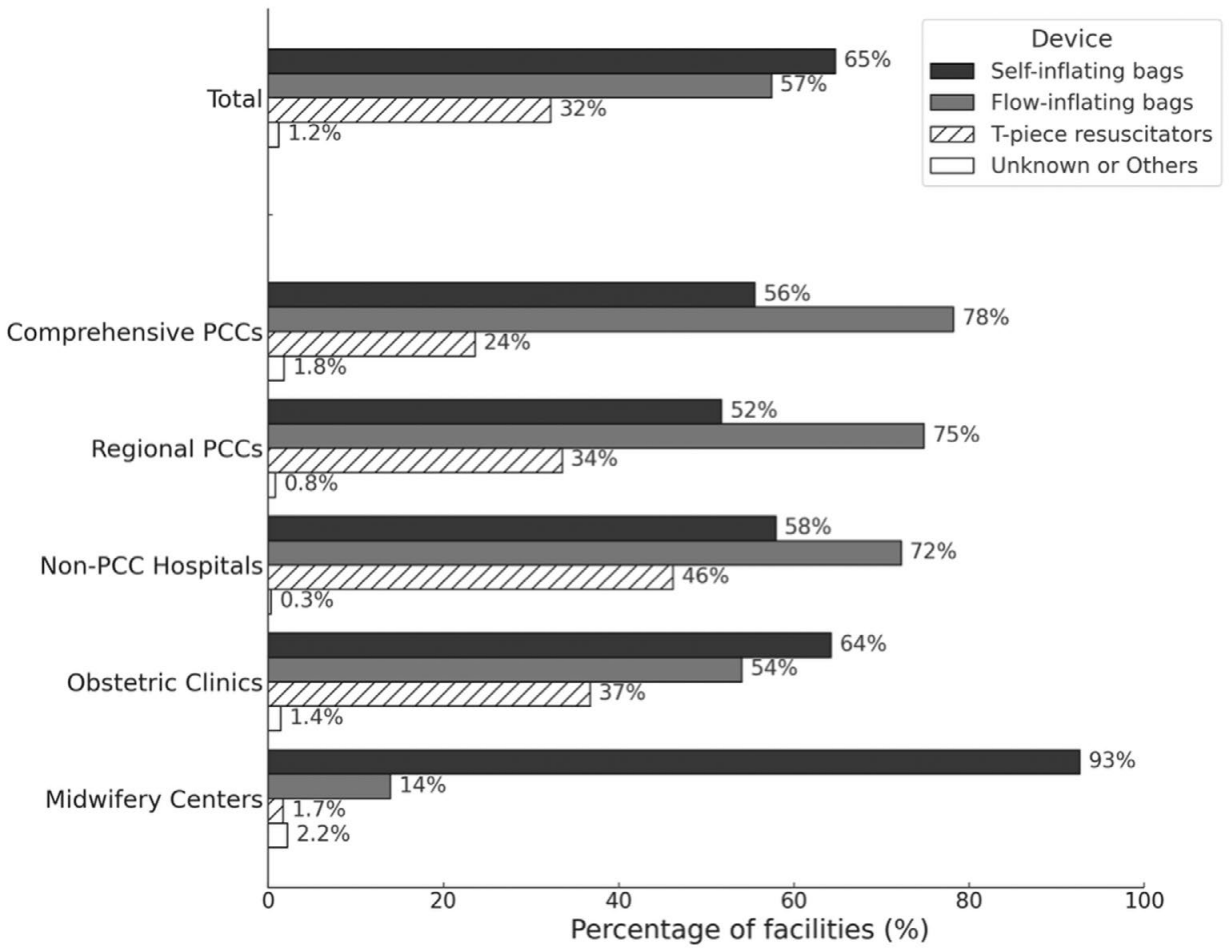
Types of equipment used for positive pressure ventilation during neonatal resuscitation based on facility type. Distribution of equipment used for positive pressure ventilation during neonatal resuscitation, stratified based on facility type. The respondents were able to select multiple devices. Equipment categories included self‐inflating bags, flow‐inflating bags, and T‐piece resuscitators. Percentages indicate the proportion of facilities reporting the use of each device type: Total (*n* = 1499), Comprehensive PCCs (*n* = 110), Regional PCCs (*n* = 242), Non‐PCC Hospitals (*n* = 342), Obstetric Clinics (*n* = 578), and Midwifery Centers (*n* = 227).

Self‐inflating bags were the most frequently used device and were used in 65% of the facilities. Flow‐inflating bags were predominantly used in higher level centers, as reported by 77% of the comprehensive PCCs and 75% of the regional PCCs. Usage was moderate in non‐PCC hospitals (72%) and obstetric clinics (54%) and low in midwifery centers (14%). T‐piece resuscitators were used in 32% of the facilities, with higher usage in non‐PCC hospitals (46%) and obstetric clinics (36%) but only in 1.7% of midwifery centers (Figure 4).

### Stabilization of neonates with respiratory distress

**FIGURE 5:**
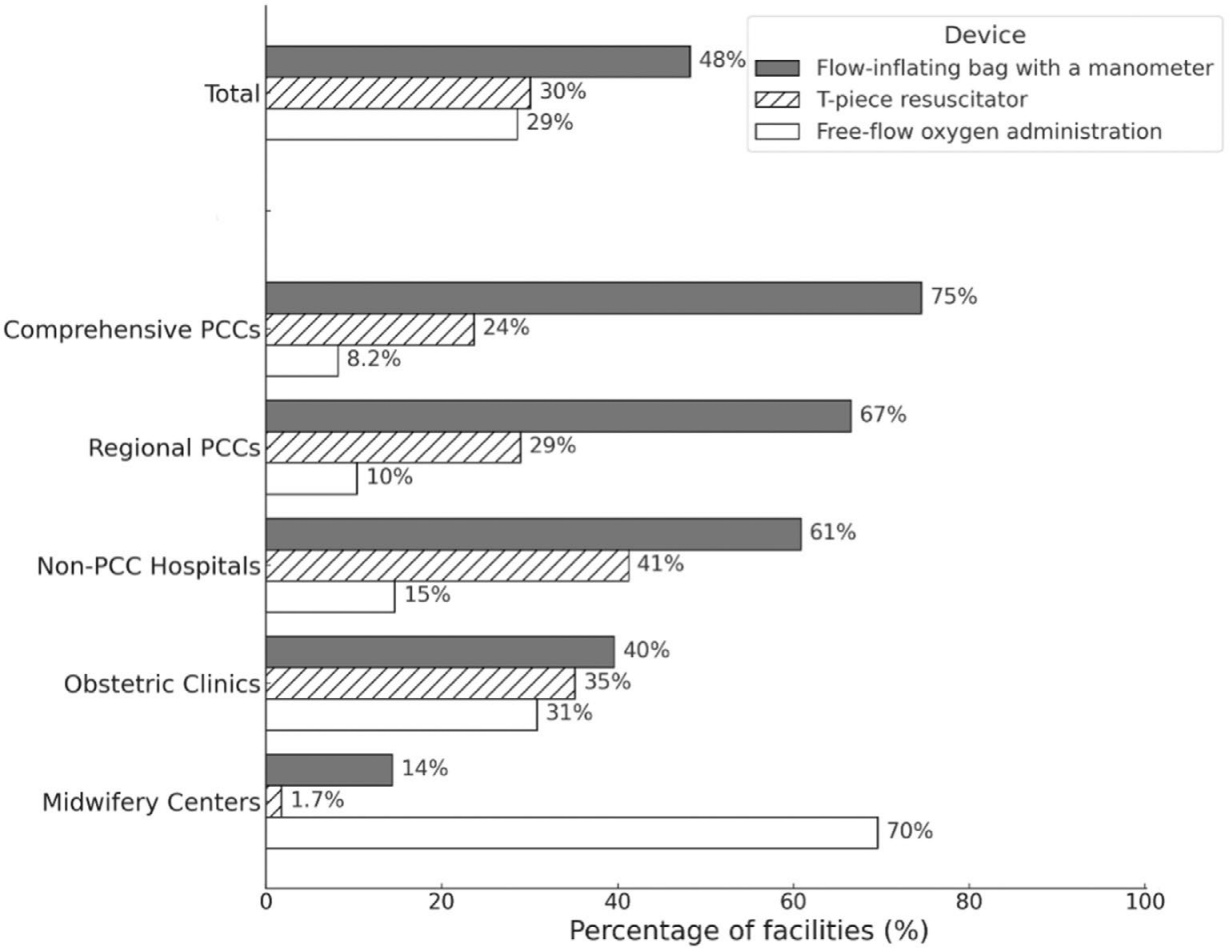
Use of continuous positive airway pressure (CPAP) and oxygen administration for term neonates with respiratory distress based on facility type. Distribution of respiratory support methods used for term neonates with respiratory distress in the delivery room, stratified based on facility type. Respondents were allowed to select multiple options. Support methods included CPAP using a flow‐inflating bag with a manometer, CPAP using a T‐piece resuscitator, and free‐flow oxygen administration. Percentages indicate the proportion of facilities reporting the use of each method within: Total (*n* = 1497), Comprehensive PCCs (*n* = 110), Regional PCCs (*n* = 240), Non‐PCC Hospitals (*n* = 340), Obstetric Clinics (*n* = 572), and Midwifery Centers (*n* = 218).

Among the facilities providing respiratory support to term neonates with respiratory distress, 48% reported using continuous positive airway pressure (CPAP) via a flow‐inflating bag with a manometer. This was followed by the use of a T‐piece resuscitator (30%) and free‐flow oxygen (29%). In midwifery centers, free‐flow oxygen was the predominant method, reported in 70% of the facilities (Figure 5).

### Supraglottic airway devices

**FIGURE 6:**
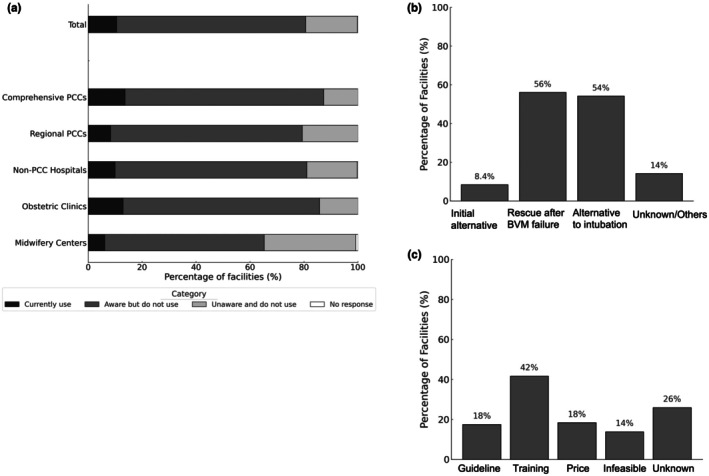
Current use and adoption intentions for supraglottic airway (SGA) devices in neonatal resuscitation. (a) Percentage of facilities with experience using SGA devices, stratified by facility type. Percentages indicate the proportion of facilities reporting current use of SGA devices among: Total (*n* = 1505), Comprehensive PCCs (*n* = 110), Regional PCCs (*n* = 242), Non‐PCC Hospitals (*n* = 342), Obstetric Clinics (*n* = 581), and Midwifery Centers (*n* = 230). (b) Reported clinical scenarios in which SGA devices were used among facilities with experience (*n* = 158). Options included: *Initial alternative* (use of an SGA device as the first alternative airway), *Rescue after bag‐mask ventilation [BVM] failure*, *Alternative to intubation* (SGA used instead of endotracheal intubation), and *Unknown/Others*. Multiple responses were allowed. (c) Reasons cited by facilities without experience using SGA devices (*n* = 1347) for potentially adopting or not adopting SGA devices in the future, assuming they are recommended in clinical guidelines. Reported reasons (abbreviated in the figure as Guideline, Training, Price, Infeasible, and Unknown) included: *Guideline recommendation*, *Training availability*, *Affordable price*, *Infeasible* (judged not feasible to introduce in practice), and *Unknown/Others*. Multiple responses were allowed.

Overall, 81% of the facilities were aware of SGA devices for neonatal resuscitation, but only 11% had actual clinical experience in using them. The usage rates remained <15% across all facility types. Among the 158 facilities with SGA experience, 56% used SGAs as a rescue measure after failed bag‐mask ventilation, and 54% used SGAs in cases of difficult intubation. The initial use of SGAs as the primary airway device was reported in 8.4% of the facilities (Figure 6a,b).

Among the 1344 facilities without clinical experience using SGAs, 18% stated that they would consider introducing SGAs if recommended by future guidelines, and 42% indicated willingness to adopt SGAs if sufficient training opportunities were available (Figure 6c).

### Umbilical cord management in preterm infants born before 28 weeks of gestation

**FIGURE 7:**
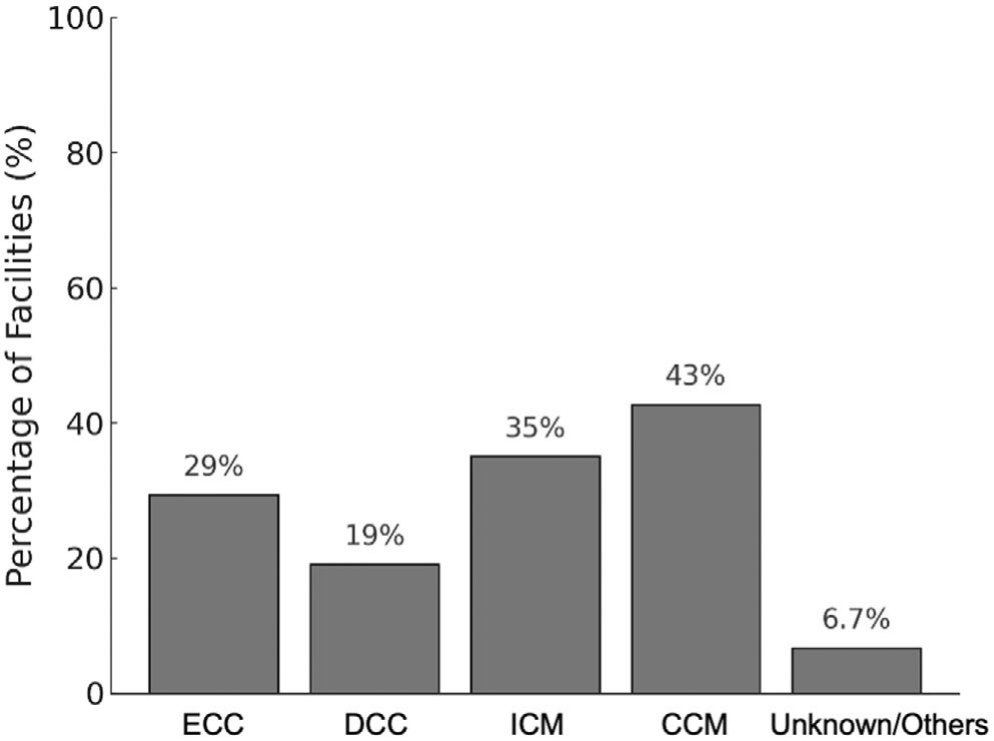
Umbilical cord management strategies for preterm infants born before 28 weeks' gestation. Reported cord management practices among 196 facilities that cared for preterm infants born before 28 weeks of gestation. Respondents were allowed to select multiple options. Options included early cord clamping (ECC, within 30 s of birth), delayed cord clamping (DCC, after 30 s), intact cord milking (ICM, milking the cord while attached to the placenta), and cut cord milking (CCM, after clamping and cutting the cord). Percentages represent the proportion of facilities reporting the use of each method.

Among the 196 facilities that manage preterm infants born before 28 weeks of gestation, various umbilical cord management practices were reported (Figure 7). Cut cord milking (CCM) was the most commonly used method, performed in 43% of the facilities. Intact cord milking (ICM) was also frequently used (35%), whereas early cord clamping (within 30 s of birth) and DCC (after 30 s) were reported in 30% and 19% of the facilities, respectively.

## DISCUSSION

This nationwide facility‐level questionnaire survey assessing neonatal resuscitation resources and practices in Japan revealed that although foundational systems, such as pulse oximetry and provider training, are widely implemented, the adoption of advanced practices, including ECG monitoring, T‐piece resuscitators, SGA devices, and updated cord management strategies, significantly varied based on facility type. These disparities were particularly evident between higher level perinatal centers and smaller clinics or midwifery centers.

### Equipment for neonatal resuscitation

Neonatal resuscitation recommends initiating with room air and adjusting the oxygen concentration based on SpO_2_ levels, ideally using pulse oximetry and an oxygen–air blender.^10^, ^13^ Our survey found that pulse oximeters were widely available across facility types. Previous nationwide surveys had already shown near‐universal adoption in hospitals and clinics, whereas midwifery centers lagged behind with coverage rates of 74% in 2013 and 72% in 2015.^11^, ^12^, ^17^ In the present survey, this increased substantially to 94%, reflecting steady improvement and a narrowing of the equipment gap between midwifery centers and other facilities.

The 2010 NCPR guidelines recommend titrating oxygen concentration with an oxygen–air blender, guided by SpO_2_ values.^17^ In the current survey, the installation rate of oxygen–air blenders was 52% overall, with higher rates in comprehensive (76%) and regional PCCs (74%) and significantly lower rates in obstetric clinics (48%) and midwifery centers (8%). In the 2013 nationwide survey, the installation rate was 83% in training hospitals (equivalent to comprehensive and regional PCCs in this study) and 41% in obstetric hospitals/clinics. In 2015, the corresponding rates were approximately 70% in training hospitals, 35% in obstetric hospitals/clinics, and 10% in midwifery centers. These findings show that while adoption was already high in perinatal centers by 2013, smaller facilities, especially midwifery centers, have consistently lagged behind through 2015 and in the present survey.^11^

ECG monitoring was emphasized in the 2015 NCPR update.^7^ Earlier nationwide surveys in 2013 and 2015 did not include ECG monitoring in their questionnaires. This study is the first to report its implementation in Japan, showing particularly low adoption in midwifery centers (5.2%). Similar to earlier trends in pulse oximetry, the widespread use of ECG monitoring may take more time, and barriers such as equipment cost and size highlight the need for further promotion through NCPR training.

### Healthcare professionals responsible for neonatal resuscitation

Previous international studies have shown wide variations in the roles of healthcare professionals involved in neonatal resuscitation and have emphasized the need for tailored training. For example, a global survey of trainees reported that physicians, nurses, and midwives played substantial roles, reflecting the multidisciplinary nature of resuscitation teams.^18^ In Japan, NCPR training has included a wide range of professionals, with midwives and nurses comprising the majority of participants.^19^

However, limited data are available regarding professionals responsible for neonatal resuscitation in clinical settings. Our nationwide survey addresses this gap and demonstrates that the allocation of responsibilities markedly differs across facility types. In higher level perinatal centers, pediatricians predominantly provide resuscitation, whereas in lower level settings, such as obstetric clinics and midwifery centers, obstetricians and midwives frequently serve as primary providers.

These findings reflect the fact that many births in Japan occur in facilities without 24‐h pediatric coverage, requiring obstetric and midwifery staff to initiate resuscitation. Although most facilities reported high completion rates for NCPR training, a small number, particularly obstetric clinics, lacked trained personnel. These results underscore the importance of strengthening training systems and clearly defining roles within interprofessional teams, particularly in nontertiary settings where pediatric specialists may not be readily available.

### Methods of positive pressure ventilation

T‐piece resuscitators offer notable advantages in neonatal resuscitation, particularly for preterm infants. A large prospective cohort study found that T‐piece use was associated with significantly lower rates of in‐hospital mortality, bronchopulmonary dysplasia, and severe intraventricular hemorrhage (IVH) than self‐inflating bags.^20^ Simulation studies have also indicated that T‐piece devices deliver a more consistent inspiratory pressure and positive end‐expiratory pressure.^21^ Based on this evidence, the CoSTR issued a weak recommendation favoring T‐piece use, despite low‐certainty evidence.^22^ However, direct comparative studies on flow‐inflating bags are lacking.

In our survey, T‐piece resuscitators were used in 32% of facilities overall, with higher adoption in non‐PCC hospitals (46%) and obstetric clinics (37%) and significantly limited use in midwifery centers (1.7%). Although adoption has increased compared to previous nationwide data, self‐inflating and flow‐inflating bags remain the predominant devices.^11^ The limited uptake of T‐piece resuscitators may be due to barriers, such as cost, lack of training, and varying levels of clinical acuity. Given that flow‐inflating bags are used in 57% of the facilities, comparative studies using T‐piece devices are required. Facility‐specific recommendations may help promote optimal device selection based on the available resources and clinical needs.

### Stabilization of neonates with respiratory distress

CPAP supports the establishment of functional residual capacity and may reduce the need for invasive ventilation. Although its benefits are well established in preterm infants,^23^, ^24^, ^25^ selective use may be advantageous in term neonates with respiratory distress despite having an adequate heart rate.^26^, ^27^ The 2022 ILCOR consensus did not make a strong recommendation for or against routine use in term and late preterm infants, citing potential benefits in reducing neonatal intensive care unit admission but also in increasing the risk of air‐leak syndromes.^22^, ^28^

In our survey, 48% of the facilities reported using CPAP via a flow‐inflating bag, 30% used a T‐piece resuscitator, and 29% used free‐flow oxygen. Notably, 70% of midwifery centers primarily relied on free‐flow oxygen, likely reflecting constraints on equipment availability, staffing, or institutional protocols.

Current NCPR guidelines permit the use of both CPAP and free‐flow oxygen.^10^

Given the inconclusive international recommendations, further evaluation of CPAP use in term and late preterm infants is warranted. Nevertheless, in facilities that manage preterm births, encouraging appropriate use of CPAP—where resources and training allow—may contribute to optimizing early respiratory support and reducing variability in practice.

### Supraglottic airway devices

The 2022 ILCOR consensus issued a weak recommendation for the use of SGA devices in newborns at 34 weeks of gestation receiving PPV, where appropriate training and equipment are available.^22^ A recent systematic review specifically evaluated the use of SGAs as an initial airway device in neonates ≥34 weeks' gestation requiring PPV. This review demonstrated high success rates for SGA insertion (>90%) and faster airway establishment compared with intubation, and also showed that the use of SGAs instead of face masks was associated with higher rates of successful resuscitation and a reduced need for subsequent intubation. However, no clear benefits were observed for oxygenation or long‐term outcomes.^29^

In our survey, 81% of the facilities were aware of SGAs, but only 10.5% had clinical experience in using them. Usage was lowest in midwifery centers and was limited to comprehensive PCCs (14%) and regional PCCs (8.3%). These findings, which are consistent with earlier data, suggest that despite growing awareness, SGAs remain underutilized in practice.^30^

Among facilities with experience, SGAs were most commonly used as rescue measures following failed bag‐mask ventilation or in cases of difficult intubation, with only a small proportion using them as initial airway devices. This pattern reflects their current positions as secondary or tertiary options for neonatal resuscitation. However, given their high success rate and ease of use, SGAs may be considered a primary option in the future, particularly in facilities where endotracheal intubation is difficult or not readily available.

Among facilities without SGA experience, 18% stated that they would consider adoption if recommended in future guidelines, and 42% indicated that they would adopt them with adequate training. These findings indicate that a lack of accessible hands‐on training is a significant barrier.

Although SGA training has not yet been integrated into the NCPR curriculum, brief manikin‐based sessions have shown high insertion success.^29^ Given their ease of use and the high success rates reported in previous studies, SGAs are particularly suitable for providers with limited airway experience and may offer practical advantages in resource‐limited settings. However, our survey revealed that both the clinical use and training opportunities for SGAs remain insufficient in Japan. Therefore, rather than recommending SGAs as a primary option at this stage, priority should first be given to establishing accessible training environments and promoting broader dissemination. Integrating SGA instruction into the NCPR curriculum and expanding simulation‐based training can promote its broader adoption. As more obstetricians and pediatricians gain exposure to SGAs during residency, their familiarity and confidence may improve. Future guidelines should address not only the clinical indications but also the institutional and educational support required for effective implementation.

### Umbilical cord management in preterm infants born before 28 weeks of gestation

In preterm infants, especially those born before 28 weeks, umbilical cord management is essential to ensure circulatory stability and adequate blood volume. Common strategies for enhancing placental transfusion include the use of DCC, ICM, and CCM. Of these, DCC provides the most robust evidence, with multiple trials showing a reduction in neonatal mortality and transfusion needs.^31^, ^32^ The ILCOR now recommends DCC for at least 60 s at all gestational ages. As this study preceded the 2024 ILCOR update,^6^ we defined DCC as ≥30, in line with the 2022 guidelines.

ICM allows rapid transfusion but may cause abrupt hemodynamic shifts, raising concerns about IVH in extremely preterm infants.^33^ The ILCOR discourages its routine use before 28 weeks due to the low certainty of evidence and possible harm.^6^ Nonetheless, over one‐third of the facilities in our study reported ICM use, underscoring the need for greater awareness of the current recommendations.

Among the 196 facilities managing infants aged <28 weeks, CCM was the most frequently used (42.9%). Although the current NCPR guidelines do not specify cord management techniques, CCM is widely adopted in Japan, likely because of its logistical feasibility and the need for prompt resuscitation. Hosono et al. showed that a mean residual blood volume of 18.3 mL/kg remained in the cord after cutting, indicating that the CCM can provide meaningful transfusion if performed properly.^34^ Unlike ICM, CCM is typically performed after breathing starts, following transfer to the resuscitation table, and thus may carry less risk of IVH. This may offer physiological benefits similar to those of DCC, making it a practical alternative when DCC is not feasible. However, the safety of CCM has not been established by robust randomized trials, and current evidence remains limited. As emphasized in the 2024 ILCOR CoSTR update, well‐designed trials are required to confirm the efficacy and safety of CCM in this vulnerable population.

### Strengths and limitations

A major strength of this study was its nationwide scope, encompassing >1500 delivery‐performing facilities across diverse healthcare settings. The high response rate (68%) and inclusion of all major facility types enhanced the generalizability of the findings. Moreover, the survey captured timely data on emerging practices, such as ECG monitoring, SGA use, and cord management, which had not been previously evaluated at the national level in Japan.

This study has certain limitations. First, it relied on self‐reported data, which may have been subject to reporting or recall biases. Second, as a cross‐sectional survey, causality and longitudinal trends could not be assessed. Third, although the survey achieved wide facility coverage, it did not capture provider‐level perspectives or variations in individual training and practice. Fourth, the response rates may differ across facility types, potentially introducing a selection bias. Finally, variability in the interpretation of technical terms may have affected some responses despite the inclusion of explanatory notes.

Despite these limitations, this study provides the most comprehensive assessment of neonatal resuscitation practices in Japan. These findings serve as a valuable foundation for future updates to national guidelines, training programs, and policy initiatives aimed at standardizing care and improving neonatal outcomes.

## CONCLUSION

Although basic NCPR systems are widely implemented in Japan, the adoption of newer practices, such as ECG monitoring and SGA use, remains limited, especially in lower level facilities. Integrating SGA instruction into the NCPR curriculum should be considered. Cord management in extremely preterm infants has shown significant variability. These findings highlight the need for updated guidelines and further studies to support safe, evidence‐based practices.

## AUTHOR CONTRIBUTIONS

Hasumi Tomita contributed to the methodology, investigation, data curation, and writing—original draft and served as the first author. Takahiro Sugiura contributed to the conceptualization, methodology, investigation, data curation, and writing—original draft and served as the corresponding author. Hitomi Arahori contributed to the methodology, investigation, visualization, data curation, and writing—original draft. Shigeharu Hosono and Tetsuya Isayama contributed to methodology, supervision, writing—review and editing, and project administration. All other authors contributed to supervision and writing—review and editing. All authors reviewed and approved the final version of the manuscript.

## FUNDING INFORMATION

This study was supported by the Neonatal Resuscitation Committee of the Japan Society of Perinatal and Neonatal Medicine.

## CONFLICT OF INTEREST STATEMENT

The authors declare no conflict of interest.

## Supporting information


Appendix S1.

Table S1.

## Data Availability

The data that support the findings of this study are available from the corresponding author upon reasonable request.
