# Supplementary material for: Nationwide survey on neonatal resuscitation across delivery facilities in Japan
Source: Pediatr Int. 2026 Feb 5;68(1):e70335. doi: 10.1111/ped.70335 (PMC12874199; doi:10.1111/ped.70335)
Supplement: Supplementary file 1 — Appendix S1. [file PED-68-e70335-s001.docx]

**Survey on Neonatal Resuscitation Equipment, Resources, and Systems**

This survey aims to assess the current status of equipment, resources, systems, and educational methods related to neonatal resuscitation. Based on the results, we hope to revise the 2025 Neonatal Resuscitation Guidelines to make them more practical and applicable.

This questionnaire is being sent to certified perinatal centers by the Japan Society of Perinatal and Neonatal Medicine, birthing facilities registered with the Japan Society of Obstetrics and Gynecology, and birthing facilities registered with the Japan Midwives Association. As such, some facilities may receive multiple notifications. If this is the case, please answer from the standpoint of the person responding to this particular questionnaire.

**1. Please select your facility’s affiliation.**

1. Perinatal (Maternal-Fetal) Care Facility certified by the Japan Society of Perinatal and Neonatal Medicine (Certification number *1: ____________) *1: https://www.jspnm.com/Senmoni/ShisetsuB.aspx
2. Perinatal (Neonatal) Care Facility certified by the Japan Society of Perinatal and Neonatal Medicine (Certification number *2: ____________) *2: https://www.jspnm.com/Senmoni/ShisetsuS.aspx
3. Other obstetric facility (not listed above)
4. Other midwifery facility (not listed above)

**2. Please select your facility type.**

1. Comprehensive Perinatal Care Center
2. Regional Perinatal Care Center
3. Hospital (other than 1 or 2)
4. Obstetric clinic
5. Independent midwifery center
6. Other

**3. Please select the prefecture your facility belongs to.** *(Radio button selection)*

**4. Please write the name of your facility.**

**5. If possible, please provide the name of the person responding to this questionnaire.**

**6. What is the occupation of the person responding to this questionnaire?**

1. Obstetrician
2. Neonatologist
3. Pediatrician
4. Anesthesiologist
5. Other physician
6. Midwife
7. Nurse
8. Other (please specify): ____________

**7. Does your facility handle deliveries?**

1. Yes
2. No *(If no, end of survey)*

**8. Are pediatricians stationed at your facility?**

1. On-site 24 hours
2. On-site during weekdays/daytime only
3. No

**9. Does your facility have an NICU (eligible for medical reimbursement)?**

1. Yes
2. No

**Section 2: Equipment and Systems for Neonatal Resuscitation**

**10. How is thermal management performed during neonatal resuscitation?** *(Multiple answers allowed)*

1. Radiant warmer
2. Closed incubator
3. Skin-to-skin contact
4. Heated mattress
5. Hot water bottle
6. Heated air blower
7. Plastic bag
8. Plastic wrap
9. Cap/hat
10. No thermal management performed
11. Unknown
12. Other (please specify): ____________

**11. Is room temperature monitored and recorded in the location where neonatal resuscitation is performed?**

1. Yes
2. No
3. Unknown
4. Other (please specify): ____________

**12. What equipment is available at the location where neonatal resuscitation is performed?** *(Multiple answers allowed)*

1. Suction device
2. Oxygen pipeline
3. Air pipeline
4. Oxygen cylinder
5. Air cylinder
6. Air compressor
7. Oxygen-air blender
8. Pulse oximeter
9. ECG monitor
10. T-piece resuscitator
11. Pediatric intraosseous needle
12. Supraglottic airway device (e.g., laryngeal mask, i-gel®)
13. Umbilical catheter
14. End-tidal CO2 detector (capnometer)
15. Respiratory function monitor (e.g., tidal volume detector)
16. Unknown
17. Other (please specify): ____________

**13. (If suction device selected in Q12) What type of suction device is commonly used?** *(Multiple answers allowed)*

1. Central suction system
2. Portable suction device
3. Bulb syringe
4. Oral suction catheter
5. Unknown
6. Other (please specify): ____________

**14. (If pulse oximeter selected in Q12) What type of sensor does the pulse oximeter use?**

1. Neonatal sensor (genuine product)
2. Neonatal sensor (non-genuine product)
3. Pediatric or adult sensor
4. Unknown

**15. What type of bag is used for positive pressure ventilation during neonatal resuscitation?** *(Multiple answers allowed; see separate sheet for items 1–3)*

1. Self-inflating bag (e.g., Ambu bag®)
2. Flow-inflating bag (e.g., Jackson-Rees®)
3. T-piece resuscitator
4. Unknown
5. Other (please specify): ____________

**16. (If flow-inflating bag selected in Q15) Is a manometer used during PPV with a flow-inflating bag?**

1. Yes
2. Depends on the situation
3. No
4. Unknown

**17. Are neonatal masks readily available in the resuscitation area?**

1. Always available
2. Not in the resuscitation area but available in the hospital
3. Not available in the hospital
4. Unknown
5. Other (please specify): ____________

**18. Are neonatal laryngoscopes readily available in the resuscitation area?**

1. Always available
2. Not in the resuscitation area but available in the hospital
3. Not available in the hospital
4. Unknown
5. Other (please specify): ____________

**19. Are neonatal endotracheal tubes available? If yes, what sizes?** *(Multiple answers allowed)*

1. 2.0 mm
2. 2.5 mm
3. 3.0 mm
4. 3.5 mm
5. 4.0 mm
6. Unknown
7. Not available
8. Other (please specify): ____________

**20. Are resuscitation medications readily available in the resuscitation area?**

1. Always available
2. Prepared as needed
3. Not available in the hospital
4. Unknown
5. Other (please specify): ____________

**21. (If answered “always available” in Q20) What medications are available?** *(Multiple answers allowed)*

1. Epinephrine (e.g., Bosmin®)
2. Normal saline
3. Sodium bicarbonate
4. Distilled water
5. 10% glucose injection
6. Unknown
7. Other (please specify): ____________

**22. Who is primarily responsible for neonatal resuscitation when interventions beyond initial stabilization are anticipated?** *(Multiple answers allowed)*

1. Obstetrician
2. Nurse
3. Midwife
4. Pediatrician
5. Anesthesiologist
6. Other physician (excluding 1, 4, and 5)
7. Unknown
8. Other (please specify): ____________

**23. Have the personnel responsible for neonatal resuscitation completed and received certification from the NCPR training course authorized by the Japan Society of Perinatal and Neonatal Medicine?**

1. All
2. Almost all
3. Some
4. None
5. Unknown
6. Other (please specify): ____________

**24. Is the NCPR resuscitation algorithm chart displayed in the delivery room?**

1. Yes (2020 version)
2. Yes (2015 or 2010 version)
3. Yes (version unknown)
4. No
5. Unknown

**25. In the delivery room, how is CPAP (Continuous Positive Airway Pressure) administered to term neonates with respiratory distress?** *(Multiple answers allowed)*

1. Flow-inflating bag with a manometer
2. T-piece resuscitator
3. Dedicated CPAP device in the delivery room
4. Only free-flow oxygen, CPAP not performed
5. Unknown
6. Other (please specify): ____________

**26. In cases of suspected severe neonatal asphyxia, is the 10-minute Apgar score recorded?**

1. Yes
2. No
3. Unknown
4. Other (please specify): ____________

**27. In the resuscitation of neonates with bradycardia due to suspected severe asphyxia, is ECG monitoring used?**

1. Almost always (80–100%)
2. Often (50–79%)
3. Sometimes (20–49%)
4. Rarely (0–19%)
5. Available in the facility but not used
6. ECG monitor available but no neonatal electrodes
7. No ECG monitor available
8. Unknown

**28. If IV drug administration is required for prolonged bradycardia during severe neonatal asphyxia, what is the first-line route of administration?**

1. Umbilical vein
2. Peripheral vein
3. Other (please specify): ____________
4. IV drug administration not performed
5. Unknown

**29. Are you aware of and do you use supraglottic airway (SGA) devices (e.g., laryngeal mask, i-gel®)?**

1. Use them
2. Aware of them but do not use
3. Not aware / do not use

**30. (If you answered “1. Use them” in Q29) What types of SGA devices are used?** *(Multiple answers allowed)*

1. With cuff
2. Without cuff
3. Unknown
4. Other (please specify): ____________

**31. (If you answered “1. Use them” in Q29) Under what circumstances are SGA devices used?** *(Multiple answers allowed)*

1. Used as the initial device (instead of bag-mask)
2. When bag-mask ventilation is ineffective
3. When intubation is difficult
4. Unknown
5. Other (please specify): ____________

**32. (If you answered “2” or “3” in Q29) If recommended in future guidelines, would you consider introducing SGA devices?** *(Multiple answers allowed)*

1. Will introduce
2. Will introduce if sufficient training is available
3. Will introduce if cost is acceptable
4. Introduction is difficult
5. Unknown
6. Other (please specify): ____________

**33. (For facilities handling infants born before 28 weeks’ gestation) How is umbilical cord management performed in these infants?** *(Multiple answers allowed)*

1. Early cord clamping (<30 seconds)
2. Delayed cord clamping (≥30 seconds)
3. Intact cord milking
4. Cut cord milking
5. Unknown
6. Other (please specify): ____________

**34. (For facilities handling infants born before 28 weeks’ gestation) What methods are used for thermal management during cord management?** *(Multiple answers allowed)*

1. Plastic bag
2. Plastic wrap
3. Heated mattress
4. Cap/hat
5. Warm linen
6. Other (please specify): ____________

35. (**For facilities that answered in the previous question that they use plastic bags or plastic wraps) Do you wipe off the infant’s moisture before using them?**

1. Yes
2. No
3. Unknown

**36. For vigorous term neonates, when is umbilical cord clamping performed?**

1. Early cord clamping (<30 seconds)
2. Late cord clamping (30–59 seconds)
3. Late cord clamping (≥60 seconds to <3 minutes)
4. Late cord clamping (until pulsation stops)
5. Unknown
6. Other (please specify specific timing): ____________

**37. Do you believe telemedicine (using video communication devices) is necessary for neonatal resuscitation in obstetric care?**

1. Strongly agree
2. Agree
3. Disagree
4. Unknown
5. Other (please specify): ____________

**38. Do you currently perform telemedicine (using video communication) during neonatal resuscitation in cooperation with affiliated institutions?**

1. Actively performed
2. Occasionally performed
3. Not performed
4. Unknown

**39. Please provide any additional comments or suggestions.**
