# Supplementary material for: Nationwide survey on neonatal resuscitation across delivery facilities in Japan
Source: Pediatr Int. 2026 Feb 5;68(1):e70335. doi: 10.1111/ped.70335 (PMC12874199; doi:10.1111/ped.70335)
Supplement: Supplementary file 2 — Table S1. [file PED-68-e70335-s002.docx]

Supplementary Table 1.

Questions 1–**9**, which collected information such as respondent name, facility name, and facility type, were excluded from the table.

**10. How is thermal management performed during neonatal resuscitation?** *(Multiple answers allowed)*]

Total facilities: 1505

| **Answer** | **Response Count** | **Proportion (%)** |
| --- | --- | --- |
| Radiant warmer | 1269 | 84.4 |
| Closed incubator | 568 | 37.8 |
| Skin-to-skin contact | 556 | 37.0 |
| Heated mattress | 444 | 29.5 |
| Hot water bottle | 268 | 17.8 |
| Heated air blower | 262 | 17.4 |
| Plastic bag | 150 | 10.0 |
| Plastic wrap | 38 | 2.5 |
| Cap/hat | 36 | 2.4 |
| No thermal management performed | 19 | 1.3 |
| Unknown | **6** | **0.4** |
| Other | 17 | 1.1 |
| No Response | **2** | **0.1** |

**11. Is room temperature monitored and recorded in the location where neonatal resuscitation is performed?**

Total facilities: 1505

| **Answer** | **Response Count** | **Proportion (%)** |
| --- | --- | --- |
| Yes | 1115 | 74.1 |
| No | 302 | 20.1 |
| Unknown | 61 | 4.1 |
| Other | 13 | 0.9 |
| No Response | 14 | 0.9 |

**12. What equipment is available at the location where neonatal resuscitation is performed?** *(Multiple answers allowed)]*

Total facilities: 1505

| **Answer** | **Response Count** | **Proportion (%)** |
| --- | --- | --- |
| Pulse Oximeter | 1472 | 97.9 |
| Suction device | 1445 | 96.1 |
| Oxygen pipeline | 1200 | 79.8 |
| ECG monitor | 843 | 56.1 |
| Oxygen cylinder | 841 | 56.0 |
| Oxygen-air blender | 778 | 51.8 |
| Air pipeline | 731 | 48.6 |
| End-tidal CO₂ detector | 595 | 39.6 |
| Air cylinder | 399 | 26.5 |
| Supraglottic airway device | 391 | 26.0 |
| Umbilical catheter | 338 | 22.5 |
| Air compressor | 306 | 20.3 |
| Respiratory function monitor | 195 | 13.0 |
| Pediatric intraosseous needle | 79 | 5.2 |
| Unknown/Others | 61 | 4.1 |
| No Response | 9 | 0.6 |

**13. (If suction device selected in Q12) What type of suction device is commonly used?** *(Multiple answers allowed)*

Total facilities: 1458

| **Answer** | **Response Count** | **Proportion (%)** |
| --- | --- | --- |
| Central suction system | 768 | 52.7 |
| Portable suction device | 628 | 43.1 |
| Bulb syringe | 172 | 11.8 |
| Oral suction catheter | 39 | 2.7 |
| Unknown/Others | 19 | 1.3 |

**14. (If pulse oximeter selected in Q12) What type of sensor does the pulse oximeter use?**

Total: 1482

| **Answer** | **Response Count** | **Proportion (%)** |
| --- | --- | --- |
| Neonatal sensor (genuine product) | 1380 | 93.0 |
| Neonatal sensor (non-genuine product) | 29 | 2.0 |
| Pediatric or adult sensor | 51 | 3.4 |
| Unknown | 22 | 1.5 |

**15. What type of bag is used for positive pressure ventilation during neonatal resuscitation?**

Total facilities: 1505

| **Answer** | **Response Count** | **Proportion (%)** |
| --- | --- | --- |
| Self-inflating bag | 970 | 64.5 |
| Flow-inflating bag | 859 | 57.1 |
| T-piece resuscitator | 481 | 32.0 |
| Unknown | 12 | 0.8 |
| No Response | 6 | 0.4 |

**16. (If flow-inflating bag selected in Q15) Is a manometer used during PPV with a flow-inflating bag?**

Total facilities: 860

| **Answer** | **Response Count** | **Proportion (%)** |
| --- | --- | --- |
| Yes | 656 | 76.3 |
| Depends on the situation | 73 | 8.5 |
| No | 73 | 8.5 |
| Unknown | 30 | 3.5 |
| Invalid Response | 28 | 3.3 |

**17. Are neonatal masks readily available in the resuscitation area?**

Total facilities: 1505

| **Answer** | **Response Count** | **Proportion (%)** |
| --- | --- | --- |
| Always available | 1475 | 98.0 |
| Not in the resuscitation area but available in the hospital | 10 | 0.7 |
| Not available in the hospital/Others/No Response | 20 | 1.3 |

**18. Are neonatal laryngoscopes readily available in the resuscitation area?**

Total facilities: 1505

| **Answer** | **Response Count** | **Proportion (%)** |
| --- | --- | --- |
| Always available | 1164 | 77.3 |
| Not in the resuscitation area but available in the hospital | 90 | 6.0 |
| Not available in the hospital | 226 | 15.0 |
| Unknown/No Response | 25 | 1.7 |

**19. Are neonatal endotracheal tubes available? If yes, what sizes?** *(Multiple answers allowed)*

Total facilities: 1505

| **Answer** | **Response Count** | **Proportion (%)** |
| --- | --- | --- |
| 2.0 mm | 426 | 28.3 |
| 2.5 mm | 857 | 56.9 |
| 3.0 mm | 1045 | 69.4 |
| 3.5 mm | 846 | 56.2 |
| 4.0 mm | 269 | 17.9 |
| Unknown | 112 | 7.4 |
| Not available | 218 | 14.5 |
| No Response | 35 | 2.3 |

**20. Are resuscitation medications readily available in the resuscitation area?**

Total facilities: 1505

| **Answer** | **Response Count** | **Proportion (%)** |
| --- | --- | --- |
| Epinephrine | 811 | 96.5% |
| Normal saline | 810 | 96.4% |
| Sodium bicarbonate | 517 | 61.5% |
| Distilled water | 609 | 72.5% |
| 10% glucose injection | 576 | 68.6% |
| Unknown | 19 | 2.3% |
| Others | 14 | 1.7% |
| No Response | 1 | 0.1% |

**22. Who is primarily responsible for neonatal resuscitation when interventions beyond initial stabilization are anticipated?** *(Multiple answers allowed)*

Total facilities: 1505

| **Answer** | **Response Count** | **Proportion (%)** |
| --- | --- | --- |
| Obstetrician | 920 | 61.1 |
| Nurse | 398 | 26.4 |
| Midwife | 878 | 58.3 |
| Pediatrician | 776 | 51.6 |
| Anesthesiologist | 82 | 5.4 |
| Other physician (excluding 1, 4, and 5) | 16 | 1.1 |
| Receiving medical staff | 22 | 1.5 |
| Unknown | 3 | 0.2 |
| No Response | 8 | 0.5 |

**23. Have the personnel responsible for neonatal resuscitation completed and received certification from the NCPR training course authorized by the Japan Society of Perinatal and Neonatal Medicine?**

Total facilities: 1505

| **Answer** | **Response Count** | **Proportion (%)** |
| --- | --- | --- |
| All | 523 | 34.8 |
| Almost all | 692 | 46.0 |
| Some | 239 | 15.9 |
| None | 25 | 1.7 |
| Unknown | 15 | 1.0 |
| Others/No Response | 11 | 0.7 |

**24. Is the NCPR resuscitation algorithm chart displayed in the delivery room?**

Total facilities: 1505

| **Answer** | **Response Count** | **Proportion (%)** |
| --- | --- | --- |
| Yes (2020 version) | 1318 | 87.6% |
| Yes (2015 or 2010 version) | 38 | 2.5% |
| Yes (version unknown) | 37 | 2.5% |
| No | 89 | 5.9% |
| Unknown | 13 | 0.9% |
| No Response | 10 | 0.7% |

**25. In the delivery room, how is CPAP (Continuous Positive Airway Pressure) administered to term neonates with respiratory distress?** *(Multiple answers allowed)*

Total facilities: 1505

| **Answer** | **Response Count** | **Proportion (%)** |
| --- | --- | --- |
| Flow-inflating bag with a manometer | 714 | 47.4 |
| T-piece resuscitator | 445 | 29.6 |
| Free-flow oxygen, CPAP not performed | 423 | 28.1 |
| Dedicated CPAP device in the delivery room | 116 | 7.7 |
| Unknown | 49 | 3.3 |
| Transfer | 17 | 1.1 |
| No experience | 13 | 0.9 |
| Others | 16 | 1.1 |
| No Response | 25 | 1.7 |

**26. In cases of suspected severe neonatal asphyxia, is the 10-minute Apgar score recorded?**

Total facilities: 1505

| **Answer** | **Response Count** | **Proportion (%)** |
| --- | --- | --- |
| Yes | 1236 | 82.1% |
| No | 139 | 9.2% |
| Unknown | 56 | 3.7% |
| No experience | 38 | 2.5% |
| Others | 22 | 1.5% |
| No Response | 14 | 0.9% |

**27. In the resuscitation of neonates with bradycardia due to suspected severe asphyxia, is ECG monitoring used?**

Total facilities: 1505

| **Answer** | **Response Count** | **Proportion (%)** |
| --- | --- | --- |
| Almost always (80–100%) | 606 | 40.3% |
| Often (50–79%) | 116 | 7.7% |
| Sometimes (20–49%) | 72 | 4.8% |
| Rarely (0–19%) | 106 | 7.0% |
| Available in the facility but not used | 76 | 5.0% |
| ECG monitor available but no neonatal electrodes | 139 | 9.2% |
| No ECG monitor available | 290 | 19.3% |
| Unknown | 52 | 3.5% |
| No /Invalid Response | 48 | 3.2% |

**28. If IV drug administration is required for prolonged bradycardia during severe neonatal asphyxia, what is the first-line route of administration?**

Total facilities: 1505

| **Answer** | **Response Count** | **Proportion (%)** |
| --- | --- | --- |
| Umbilical vein | 377 | 25.0 |
| Peripheral vein | 375 | 24.9 |
| IV drug administration not performed | 531 | 35.3 |
| Unknown | 139 | 9.2 |
| Endotracheal administration | 22 | 1.5 |
| Unsettled | 23 | 1.5 |
| No Response | 38 | 2.5 |

**29. Are you aware of and do you use supraglottic airway (SGA) devices (e.g., laryngeal mask, i-gel®)?**

Total facilities: 1505

| **Answer** | **Response Count** | **Proportion (%)** |
| --- | --- | --- |
| Use them | 158 | 10.5 |
| Aware of them but do not use | 1055 | 70.1 |
| Not aware / do not use | 289 | 19.2 |
| No /Invalid Response | 3 | 0.2 |

**30. (If you answered “1. Use them” in Q29) What types of SGA devices are used?** *(Multiple answers allowed)*

Total facilities: 158

| **Answer** | **Response Count** | **Proportion (%)** |
| --- | --- | --- |
| With cuff | 63 | 39.9 |
| Without cuff | 89 | 56.3 |
| Unknown | 12 | 7.6 |
| No Response | 3 | 1.9 |

**31. (If you answered “1. Use them” in Q29) Under what circumstances are SGA devices used?** *(Multiple answers allowed)*

Total facilities: 158

| **Answer** | **Response Count** | **Proportion (%)** |
| --- | --- | --- |
| Used as the initial device (instead of bag-mask) | 13 | **8.4** |
| When bag-mask ventilation is ineffective | 87 | **56.1** |
| When intubation is difficult | 84 | **54.2** |
| Unknown/Others | 22 | **14.1** |
| No Response | 3 | **0.2** |

**32. (If you answered “2” or “3” in Q29) If recommended in future guidelines, would you consider introducing SGA devices?** *(Multiple answers allowed)*

Total facilities: 1344

| **Answer** | **Response Count** | **Proportion (%)** |
| --- | --- | --- |
| Will introduce | 235 | 17.5 |
| Will introduce if sufficient training is available | 559 | 41.6 |
| Will introduce if cost is acceptable | 247 | 18.4 |
| Introduction is difficult | 186 | 13.8 |
| Unknown | 348 | 25.9 |
| No Response | 3 | 0.2 |

**33. (For facilities handling infants born before 28 weeks’ gestation) How is umbilical cord management performed in these infants?** *(Multiple answers allowed)*

Total facilities: 194

| **Answer** | **Response Count** | **Proportion (%)** |
| --- | --- | --- |
| Early cord clamping (<30 seconds) | 57 | 29.4 |
| Delayed cord clamping (≥30 seconds) | 37 | 19.1 |
| Intact cord milking | 68 | 35.1 |
| Cut cord milking | 83 | 42.8 |
| Unknown | 13 | 6.7 |
| No Response | 0 | 0.0 |

**34. (For facilities handling infants born before 28 weeks’ gestation) What methods are used for thermal management during cord management?** *(Multiple answers allowed)*

Total facilities: 194

| **Answer** | **Response Count** | **Proportion (%)** |
| --- | --- | --- |
| Plastic wrap | 141 | 72.7 |
| Plastic bag | 25 | 12.9 |
| Warm linen | 46 | 23.7 |
| Heated mattress | 31 | 16.0 |
| Cap/hat | 29 | 14.9 |
| Not used | 7 | 3.6 |
| Unknown | 4 | 2.1 |
| No Response | 11 | 5.7 |

**35. (For facilities that answered in the previous question that they use plastic bags or plastic wraps) Do you wipe off the infant’s moisture before using them?**

Total facilities: 155

| **Answer** | **Response Count** | **Proportion (%)** |
| --- | --- | --- |
| Yes | 141 | 72.7 |
| No | 25 | 12.9 |
| Unknown | 46 | 23.7 |
| No Response | 31 | 16.0 |

**36. For vigorous term neonates, when is umbilical cord clamping performed?**

Total facilities: 1505

| **Answer** | **Response Count** | **Proportion (%)** |
| --- | --- | --- |
| Early cord clamping (<30 seconds) | 847 | 56.3 |
| Late cord clamping (30–59 seconds) | 282 | 18.7 |
| Late cord clamping (≥60 seconds to <3 minutes) | 114 | 7.6 |
| Late cord clamping (until pulsation stops) | 174 | 11.6 |
| Depends on the situation | 12 | 0.8 |
| Unknown | 39 | 2.6 |
| No Response | 37 | 2.5 |

**37. Do you believe telemedicine (using video communication devices) is necessary for neonatal resuscitation in obstetric care?**

Total facilities: 1505

| **Answer** | **Response Count** | **Proportion (%)** |
| --- | --- | --- |
| Strongly agree | 174 | 11.6 |
| Agree | 585 | 38.9 |
| Disagree | 293 | 19.5 |
| Unknown | 435 | 28.9 |
| Others | 10 | 0.7 |
| No Response | 8 | 0.5 |

**38. Do you currently perform telemedicine (using video communication) during neonatal resuscitation in cooperation with affiliated institutions?**

Total facilities: 1505

| **Answer** | **Response Count** | **Proportion (%)** |
| --- | --- | --- |
| Actively performed | 26 | 1.7 |
| Occasionally performed | 33 | 2.2 |
| Not performed | 1389 | 92.3 |
| Unknown/Others | 50 | 3.3 |
| No Response | 7 | 0.5 |
